# Haplotype-resolved assembly of the mule duck genome using high-fidelity sequencing technology

**DOI:** 10.1371/journal.pone.0305914

**Published:** 2024-07-01

**Authors:** Tiandong Che, Jing Li, Xiaobo Li, Zhongsi Wang, Xuemei Zhang, Weifei Yang, Tao Liu, Yan Wang, Kaiqian Wang, Tian Gao, Guangqiang Shen, Wanling Qiu, Zhimin Li, Wenguang Zhang

**Affiliations:** 1 College of Life Science, Inner Mongolia Agricultural University, Hohhot, China; 2 Annoroad Gene Technology Co., Ltd, Beijing, China; 3 Institute of Animal Genetics and Breeding, College of Animal Science and Technology, Sichuan Agricultural University, Chengdu, Sichuan, China; Central University of Kerala, INDIA

## Abstract

Mule duck is vitally important to the production of global duck meat. Here, we present two high-quality haplotypes of a female mule duck (haplotype 1 (H1):1.28 Gb, haplotype 2 (H2): 1.40 Gb). The continuity (H1: contig N50 = 14.90 Mb, H2: contig N50 = 15.70 Mb) and completeness (BUSCO: H1 = 96.9%, H2 = 97.3%) are substantially better than those of other duck genomes. We detected the structural variations (SVs) in H1 and H2. We observed a positive correlation between autosome length and the number of SVs. Z chromosome was some deficient in deletions and insertions, but W chromosome was some excessive. A total of 1,451 genes were haplotype specific expression (HSEs). Among them, 737 specifically expressed in H1, and 714 specifically expressed in H2. We found that H1 and H2 HSEs tended to be involved in similar biological processes, such as myometrial relaxation and contraction pathways, muscle structure development and phosphorylation. Our haplotype-resolved genome assembly provides a powerful platform for future functional genomics, molecular breeding, and genome editing in mule duck.

## Introduction

The global production of duck meat has experienced a significant surge in recent times. The primary global producer of duck meat is Asia, particularly due to the Pekin duck [1]. China accounts for approximately 84% of the total Asian duck production [2]. In Europe, Pekin duck dominates in most countries, especially in northern and central eastern regions. In the Mediterranean countries such as France and Italy, mule and Muscovy ducks are more prevalent [3], contributing to around 11% of the worldwide duck meat supply [2].

Mule ducks, which are sterile hybrids resulting from the crossbreeding of male Muscovy ducks (*Cairina moschata*) [4] and female Pekin ducks (*Anas platyrhynchos*), demonstrate a remarkable tolerance towards adverse environmental conditions, temperature fluctuations, and diseases. They exhibit rapid growth rates, efficient feed conversion, and superior meat quality. Male mule ducks are particularly preferred for fatty liver production [5, 6]. Indeed, mule ducks benefit from heterosis effects and inherit specific qualities from each parent species, including increased feed intake capacity [7]. Recent research shows that mule ducks are characterized by lower water and higher fat and zinc content in leg muscle compared to Muscovy ducks [2].

With the continuous development of long-read sequencing technology, such as PacBio and Oxford Nanopore, researchers are able to assemble high-quality genomes for subsequent studies. Among them, the introduction of HiFi technology enables researchers to decode complex regions on the genome, such as telomeres and centromeres [8], more accurately. It has been proven that HiFi technology performs well in terms of continuity and integrity in genome assembly, and is widely used in areas such as telomere-to-telomere (T2T) genomes [9, 10], haplotype-resolved genomes [11, 12], and gap-free genome assemblies [13–15].

Here, we provide a haplotype-resolved female mule duck genome, which will be a valuable resource for genetic breeding programs with mule ducks, especially in meat production.

## Materials and methods

### Animals and sample collection

Three 60-days old healthy female mule ducks were examined in this study. The muscle of leg was rapidly dissected from each carcass and immediately frozen in liquid nitrogen. All samples were stored at −80˚C until total RNA extraction. Animals were humanely killed to ameliorate suffering by intravenous injection with 2% pentobarbital sodium (25 mg/Kg). All experimental procedures and sample collection in this study were approved by the Institutional Animal Care and Use Committee (IACUC) of Sichuan Agricultural University, under permit No. DKY-B20141401.

### Genome sequencing and assembly

The mule duck genome was sequenced and assembled using DNA from a female individual at 60 days old (one of the three female mule ducks used for total RNA extraction). To generate PacBio HiFi data, high-molecular-weight genomic DNA from the muscle of mule duck was extracted using FineOut Universal Animal and Plant Genome Extraction reagent (solution type). DNA integrity was assessed using Femto Pulse. Megaruptor was used to interrupt 6.5 μg DNA for genome fragments, after which AMPure PB beads were used for purification. Two SMRT bell libraries were constructed using the Pacific Biosciences SMRT bell express template prep kit 2.0. The constructed libraries were selected for fragment size on the BluePippin™ system, constructed with insertion fragment size of 15 Kb, then subjected to primer annealing and bound the SMRTbell template to the polymerase using a DNA/polymerase binding kit. The library was sequenced on Sequel IIe platforms for 30 hours.

Haplotype-resolved genome assembly contigs were generated using hifiasm software (v0.15.2) [16] on HiFi reads, and the resulting GFA format files were converted to FASTA files using gfatools (see URLs). The high base quality of the HiFi contigs [17] precluded polishing; however, we used RagTag (v2.0.1) [18], with Muscovy duck (*C*. *moschata*) and Pekin duck (*A*. *platyrhynchos*) reference genomes and HiFi reads, to correct potential misassemblies in haplotype contigs before assigning them to chromosomes via the RagTag scaffold command based on both reference genomes. Based on these, we should be able to determine which haplotypes come from which parent. Completeness of haplotype-resolved genome assemblies was evaluated using Benchmarking Universal Single-Copy Orthologs (BUSCO v4.1.2, aves_odb10) software [19].

### RNA-seq and genome annotation

*RNA-seq*. Total RNA was isolated using a standard Trizol (Invitrogen) protocol. Genomic DNA was removed using DNaseI. Three cDNA libraries for paired-end 150 bp sequencing were prepared using dUTP protocols. Libraries were sequenced on Illumina NovaSeq 6000 platform. More than 10 Gb of high quality data were obtained per library.

*Gene structure prediction*. Gene structure prediction was performed using a comprehensive approach that integrated three strategies. Firstly, gene structure was predicted based on evidence supported by transcriptome data from RNA-seq analysis, utilizing cDNA sequence and genome alignment in conjunction with PASA (v2.1) [20]. Secondly, homology evidence was employed by comparing protein-coding sequences of related species to the mule duck genome using Blast (v2.2.28) [21] and Genewise (v2.2.0) [22]. Lastly, ab initio predictions generated by AUGUSTUS (v3.3) [23] and SNAP (v2013-11-29) [24] were utilized for gene structure prediction as well. Finally, these results were integrated into a non-redundant and more comprehensive gene set using GETA (v2.5.4), as described in the provided URLs.

*Gene function annotation*. Various functional databases, including Swissprot (release 2018_12) [25], NT, NR, PFAM (v35.0) [26], eggnog (v3.0) [27], GO [28], and KEGG [29], were employed for the prediction of gene set functions.

*Noncoding RNA prediction*. The identification of noncoding RNAs involved the utilization of tRNAScan-SE (v1.3.1) [30] for tRNA detection, while other types of ncRNAs were identified through a search against the Rfam (v12.0) database [31].

*Characterization of repetitive sequences*. Characterization of repetitive sequences was performed using two integrated strategies. Firstly, homology evidence was utilized by employing RepeatMasker (v4.0.7) and Repeatproteinmask (v1.36) [32], along with the RepBase (v28.06) database [33], to predict sequences that exhibited similarity to known repetitive sequences. Secondly, an ab initio prediction approach was employed where repeat families were initially identified de novo and classified using RepeatModeler (v1.0.10). The resulting repeat library generated by RepeatModeler (v1.0.10) was further analyzed using RepeatMasker (v4.0.7) [32] to uncover additional repeats in the genome, while TRF [34] was employed for identifying tandem repeat sequences.

### Assembly-driven detection of structural variation

The two haplotype-resolved genomes were aligned to their respective reference genomes by minimap2 v2.21 [35]. SVIM [36] was adopted to detect SVs, including deletions (DEL), insertions (INS), duplications (DUP) and inversions (INV). We filtered out SVs which were smaller than 50 bp. We used the lm function to perform regression analysis between chromosome length and SV number by R.

### Identification of haplotype differentially expressed genes

The RNA-seq reads from muscle tissue were generated using three biological replicates. Subsequently, the reads were aligned against the coding sequence (CDS) using Bowtie (v1.3.1) [37], and only the best alignment was retained for each read. FPKM values were estimated utilizing the RSEM program (v1.3.3) [38]. To investigate differences in expression between alleles, we employed the DESeq2 package (v1.40.2) [39]. Differentially expressed genes were selected based on a fold change (FC) > 2 with Benjamini-Hochberg adjusted p-values < 0.05 as criteria for significance assessment.

### Calculation of Shannon entropy

We calculated the Shannon entropy (*H*) using the formula described in the original paper [40] as:

$$H_{g}=\sum_{1\leq t\leq N}-P_{t/g}\log_{2}(P_{t/g})$$

where *P*_*t/g*_ is the relative expression of a gene g in a haplotype t relative to its expression given in N haplotypes. This value has units of bits ranging from zero, indicating genes expressed in a single haplotype, to log2(N), indicating genes expressed uniformly in all haplotypes. Q value was calculated as:

$$Q_{g/t}=H_{g}-\log_{2}(P_{t/g})$$

It could be used to determine that gene g has specific expression in haplotype.

### Analysis of haplotype specific expression genes

We defined the haplotype specific expression genes (HSEs) using the following criteria: i) the genes with Benjamini-Hochberg adjusted p-values < 0.01; ii) the Shannon entropy (*H*) of the genes equal to zero. Functional enrichment analyses for genes were performed by Metascape [41].

## Results

### Genome sequencing and assembly

We sequenced the genome of a female mule duck, an F1 hybrid with Muscovy duck as the sire (paternal contributor) and domestic duck as the dam (maternal contributor), by using the PacBio Sequel IIe platform, generating 73.10 Gb of HiFi reads (~73-fold coverage, S1 Table in S1 File). We performed *de novo* haplotype-resolved genome assembly using hifiasm (v0.15.2) [16] period. This yielded 729 contigs in haplotype 1 (H1, Muscovy duck genetic, with Z chromosome sequences) and 1,422 contigs in haplotype 2 (H2, Mallard genetic, with W chromosome sequences). The genome size of the final assembly of H1 and H2 was 1.28 Gb (N50 of 14.90 Mb) and 1.40Gb (N50 of 15.70 Mb), respectively (Table 1). The continuity of each mule duck haplotype was substantially higher than that of the parental origin assemblies of the Muscovy duck (*C*. *moschata*, KizCaiMos1.0) [42] and Pekin duck (*A*. *platyrhynchos*, ZJU1.0) [43], demonstrating the advantage of HiFi data in phased genome assembly (Table 1). We evaluated the completeness of our phased genomes using BUSCO [19]. H1 showed over 96.9% coverage of the embryophyte orthologous gene set, whereas H2 showed 97.3% coverage (Table 1 and S2 Table in S1 File).

**Table 1:** Genome features of the two haplotypes.

Genomic features	Assembled genome size (Gb)^a^	Number of Contigs	Contig N50 (Mb)^b^	Largest contig length (Mp)	BUSCO	Number of protein-coding genes	Repeat content (%)
**H1 (Muscovy duck)**	**1.28**	**729**	**14.90**	**62.29**	**96.90%**	**18,002**	**20.44**
**H2 (Pekin duck)**	**1.40**	**1,422**	**15.70**	**55.58**	**97.30%**	**19,385**	**22.07**
***C*. *moschata* (Muscovy duck)[KizCaiMos1.0]**	**1.1**	**-**	**11.81**	**53.95**	**94.30%**	**15,829**	**10.3(TE)** ^**c**^
***A*. *platyrhynchos* (Pekin duck)[ZJU1.0]**	**1.2**	**1,645**	**5.68**	**28.52**	**94.20%**	**16,836**	**23.92**

^a^ The fragments of the ungapped genome assembly.

^b^ N50 (50% of the genome is in fragments of this length or longer) of genome assembly was calculated using the fragments longer than 500 bp.

^c^ TE are Transposable elements.

**Fig 1:**
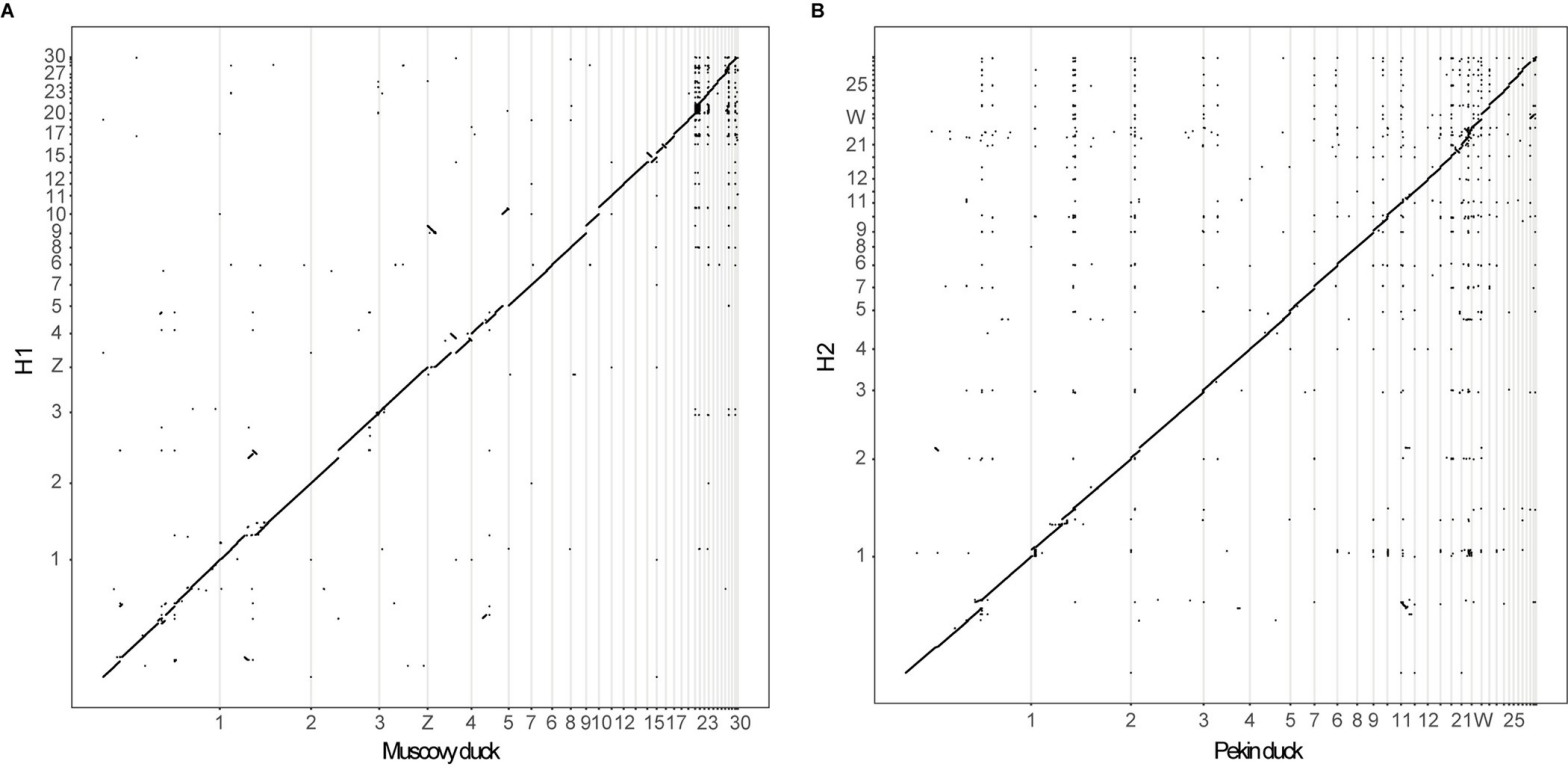
Dot plot showing the syntenic relationships between (A) H1 and Muscovy duck, (B) H2 and Pekin duck. The X-axis represents the Muscovy duck and Pekin duck, and Y-axis represents the H1 and H2. A diagonal straight line indicates synteny among the haplotypes.

The contigs were scaffolded into a chromosome-level sequence based on the reference genome sequences (Muscovy duck and Pekin duck) guided strategy by RagTag (v2.0.1) [18], resulting in 35 autosomes plus Z chromosomes in H1 and 31 autosomes plus W chromosomes in H2. We aligned the chromosome-level sequences of H1 and H2 to Muscovy duck (*C*. *moschata*) and Pekin duck (*A*. *platyrhynchos*) respectively, and found overall high similarity between them at the sequence level (Fig 1). We also aligned H1 and H2 to each other, which revealed a highly collinear relationship (S1 Fig in S1 File). In this alignment, we found that the chrZ in H1 aligned to chrW in H2, which implied that these two sex chromosomes exhibit a high degree of homology (S3 Table in S1 File). In addition, we have observed the presence of collinearity between certain other chromosomes, such as chr10, 13, 18, 19, 24, 25 (S3 Table in S1 File).

### Gene and repeat annotations

We proceeded to annotate the genome using a comprehensive strategy combining evidence-based and ab initio gene predictions (see “Materials and methods”). Based on the gene structure, 29,928 gene models were predicted in H1 with an average gene length of 18627.76 bp, whereas 31,661 gene models were predicted in H2 with an average gene length of 18211.15 bp (S4 Table in S1 File). Combining the evidence from mRNA sequencing (RNA-seq) data, homologous and ab initio gene predictions, we obtained 18,002 and 19,385 protein-coding genes in H1 and H2, respectively (Table 1), which were integrated using GETA (v2.4.14) (see URLs). Among them, functional annotation showed that 97.86% and 97.60% of the protein-coding genes in H1 and H2 matched known proteins in public databases (S5 Table in S1 File). In addition, we also identified small noncoding RNAs (ncRNAs) across the two haplotypes, including microRNA (miRNA), ribosomal RNAs (rRNAs), transfer RNAs (tRNAs) and small nuclear RNA (snRNA) (S6 Table in S1 File). The exon number, gene length, coding sequence (CDS) length, exon length, and intron length were similar across other related species (S2 Fig in S1 File).

Our assembly contains 20.44% and 22.07% of repetitive sequences in H1 and H2, respectively (Table 1). We identified 685,186 and 761,147 copy number of repeat elements in H1 and H2, among which long terminal repeat endogenous retroviruses (LTR/ERV) were abundant, making up 3.74% and 3.64% of the two haplotype genomes, and LTR/Gypsy elements were particularly plentiful, accounting for 0.63% and 1.08% of the genomic content, respectively (S7 Table in S1 File). We observed that LINE accounted for the highest proportion (5.13%) in H2, which implies the a high prevalence in mallard genome.

### Characteristics of structural variation

We detected a total of 23,229 and 35,893 structural variations (SVs) in H1 and H2 genomes against their reference genomes, including 8,454 and 17,959 deletions, 14,719 and 17,759 insertions, 27 and 103 duplications, 29 and 72 inversions. Deletions and insertions were the majority type of SVs, and this was also the case on each chromosome (S3 Fig in S1 File). The H2 has more deletions, while the H1 has more insertions.

**Fig 2:**
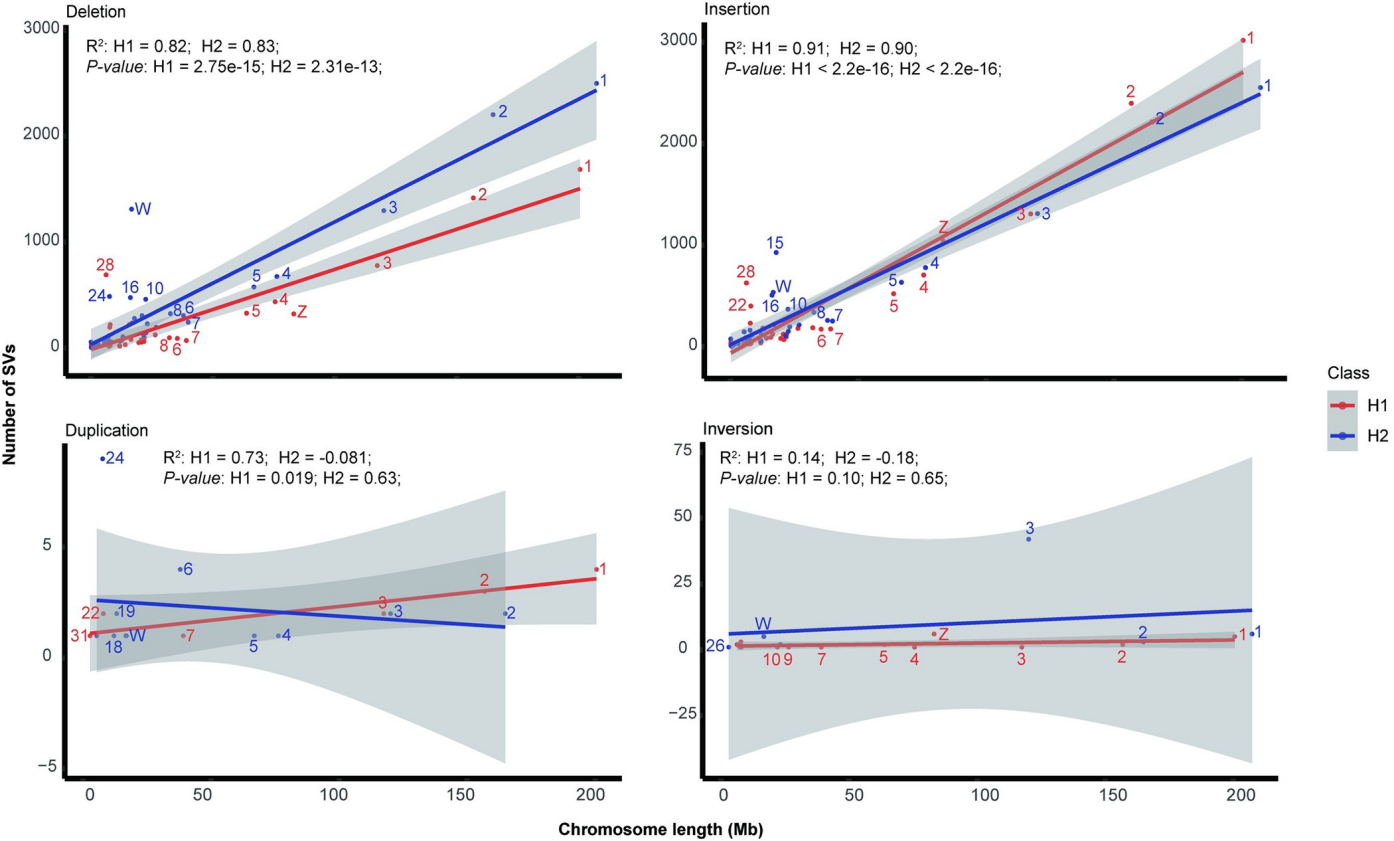
Regression of chromosome length and the number of structural variations (SVs).

In order to explore the relationship between chromosome length and the number of structural variations, we conducted a regression analysis. In autosomes, we observed a positive correlation between the length of chromosomes and the number of SVs (Fig 2). Most of the chromosomes fell within or around the 99% confidence interval. Compared with the autosomes, the Z and W chromosomes exhibited a certain degree of deviation, especially in deletions and inversions. The Z chromosome was somewhat deficient in deletions and insertions, but the W chromosome was somewhat excessive. This phenomenon suggests that there might be differences in the sensitivity of sex chromosomes to SVs, which could be related to their functional conservation.

### Haplotype specific expression genes

Given that the female mule duck was an F1 hybrid offspring, our haplotype-resolved genomes enabled the exploration of genes with allelic imbalance in expression. However, since muscle tissue in poultry is a major economic trait, leg muscle was chosen to demonstrate gene phasing to explore haplotype specific expression. We applied mRNA sequencing (RNA-seq) to the left leg muscle of three female mule ducks, and quantified the allelic expression levels of H1 and H2. We used the two haplotypes to quantify and obtain FPKM expression values of the protein-coding genes. An average of ~12,184 genes were as expressed (with an FPKM value > 0.1) [44] in each haplotype from three female mule ducks, and 14,919 genes were expressed in all six haplotypes (from three female mule ducks). We used the R package DESeq2 (v1.40.2) [39] to perform the differential expression analysis to compare the two haplotypes. Genes with a fold change (FC) > 2 and Benjamini-Hochberg adjusted p values < 0.05 were classified as differentially expressed (DE). Based on this, we found 2,567 differential expression genes between H1 and H2 (S8 Table in S1 File).

**Fig 3:**
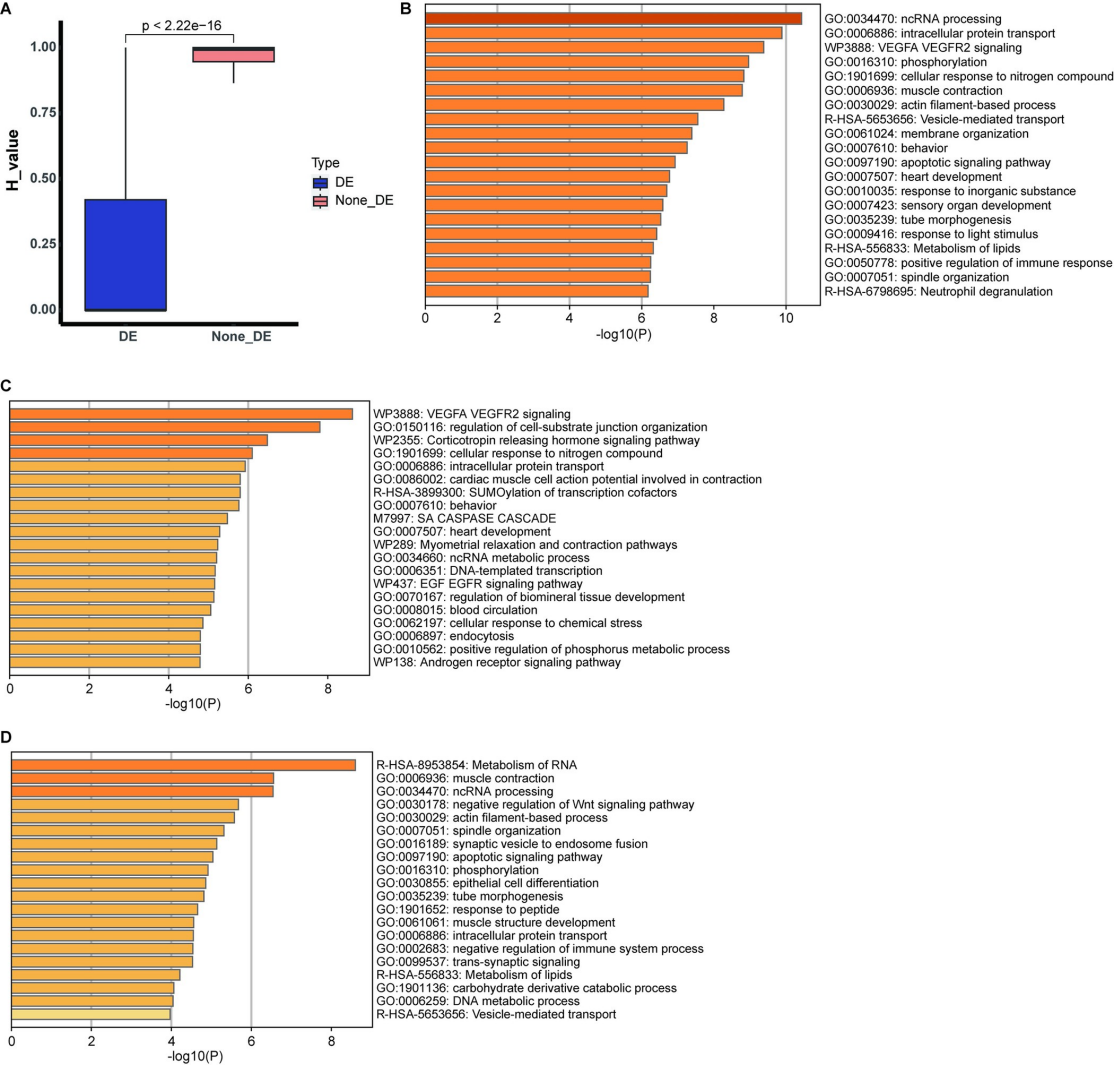
Shannon entropy (*H*) value between DE and non-DE genes and functional categories of HSEs. (A) Boxplot of Shannon entropy (H) value between DE and non-DE genes. (B) Functional categories of all HSEs. (C) Functional categories of H1 specific expression genes. (D) Functional categories of H2 specific expression genes.

To identify the haplotype-specific expression genes (HSEs), we calculated the Shannon entropy (*H*) value as a measure of the specificity of gene expression across haplotypes. We found that the DEs showed increased haplotype specificity compared with none DEs (Fig 3A). We selected the HSEs (see methods), and obtained 1,451 HSEs (S9 Table in S1 File). Among them, 737 specifically expressed in H1, and 714 were specifically expressed in H2 (refer to S10 and S11 Tables in S1 File). Functional enrichment analysis showed that the HSEs were mainly enriched for phosphorylation (GO:0016310), muscle contraction (GO:0006936) and actin filament-based process (GO:0030029) (Fig 3B), which fit with the biological function of muscle tissue. Besides, we found that H1 and H2 HSEs tended to be involved in similar biological processes. HSEs in H1 were enriched for myometrial relaxation and contraction pathways (WP289) and EGF and EGFR signaling pathway (WP437) (Fig 3C). HSEs in H2 were enriched for muscle contraction (GO:0006936), actin filament-based process (GO:0030029), muscle structure development (GO:0061061) and phosphorylation (GO:0016310) (Fig 3D).

## Discussion

In this study, we assembled a chromosome-scale haplotype-resolved female mule duck genome based on HiFi sequencing data. Both the sequence continuity (contig N50) and genome quality (base accuracy and completeness) were substantially higher than those of the previously released duck genomes (Muscovy duck and Pekin duck). Our results indicated the advantages of HiFi reads on de novo genome assembly and provided a valuable resource for future genetic breeding programs.

Additionally, we attempted to detect structural variations (SVs) using an assembly-based approach and compared them with chromosome length [45]. Consistent with previous studies, we observed a deviation between the number of SVs in sex chromosomes and chromosome length. In the context of normal natural selection, the number of SVs should be linearly correlated with the length of the chromosome. Our results indicated that the Z chromosome had fewer SVs, while the W chromosome had more SVs. This phenomenon suggested that the Z and W chromosomes may have undergone different intensities of purifying selection.

In addition, this chromosome-scale haplotype-resolved female mule duck genome will enable the characterization of haplotype specific genes and their functions. Based on the differentially expressed genes, we added Shannon entropy (*H*) value to screen for genes with haplotype-specific expression (HSEs). Through the functional enrichment analysis, we discovered that HSEs were more involved in pathways related to energy metabolism and muscle contraction, which was consistent with the biological characteristics of muscle tissue. Functional analysis of H1 and H2-specific expression genes yielded similar results, further confirming the biological function of muscle.

In summary, our research provides the haplotype-resolved genome of a hybrid species, which will serve as a reference for future genomic studies on other hybrid species. However, mapping some highly repetitive regions, such as those related to the telomere and centromere, remains a challenge in mapping a diploid mule duck genome. Efforts have tried to address these challenges. For example, the first human telomere-to-telomere (T2T) genome (CHM13) [9, 46] has been constructed. Additionally, a draft of human pangenome has been published [47], which implies that a more comprehensive, diverse and accurate genomic age is forthcoming.

## Supporting information

S1 File (ZIP)
