# Supplementary figures and images for: Haplotype-resolved assembly of the mule duck genome using high-fidelity sequencing technology
Source: PLoS One. 2024 Jul 1;19(7):e0305914. doi: 10.1371/journal.pone.0305914 (PMC11216606; doi:10.1371/journal.pone.0305914)

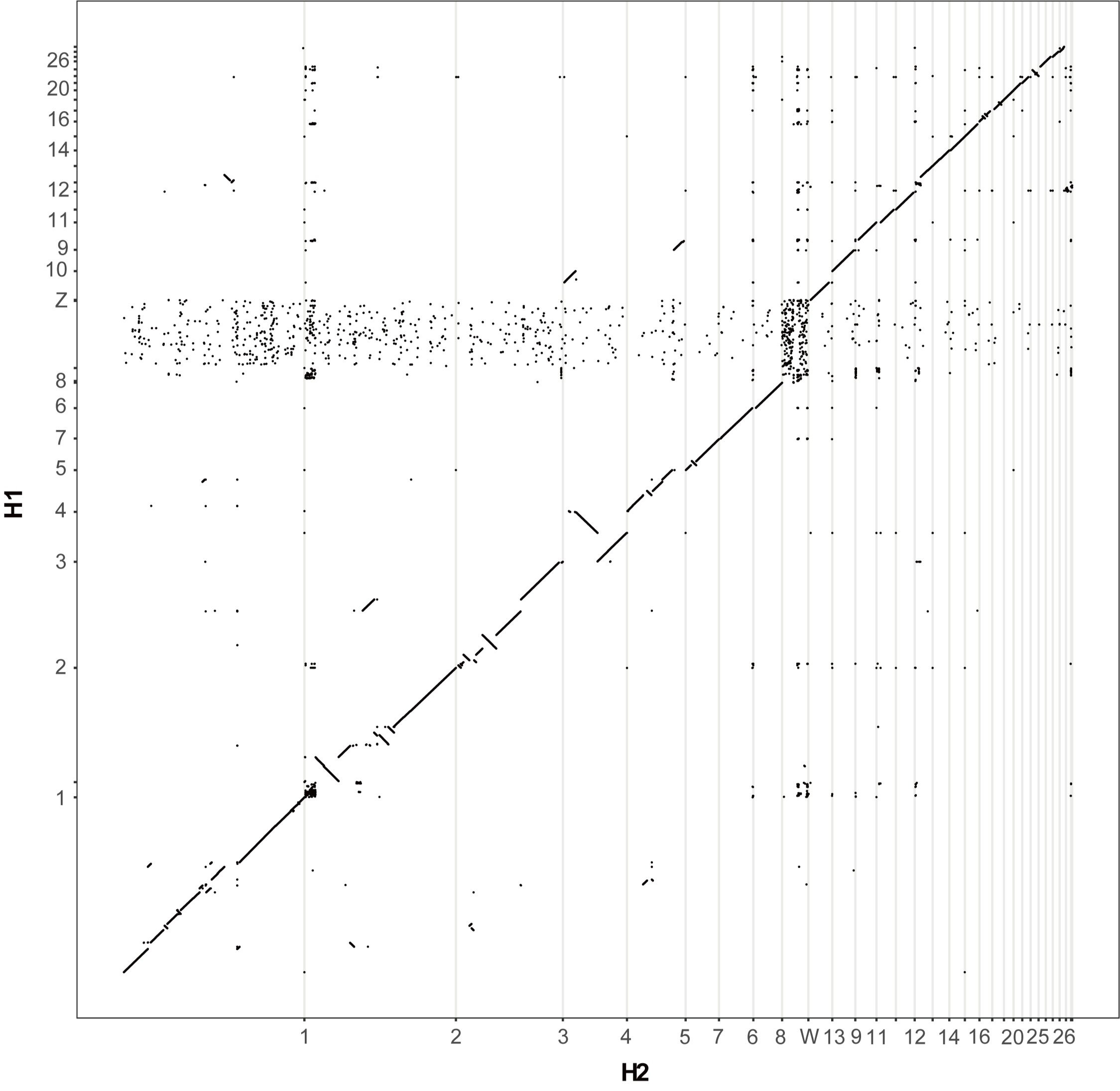

Supplement: S1 File — (ZIP) [file pone.0305914.s001.zip › Supporting_Information/S1_Fig.tif]

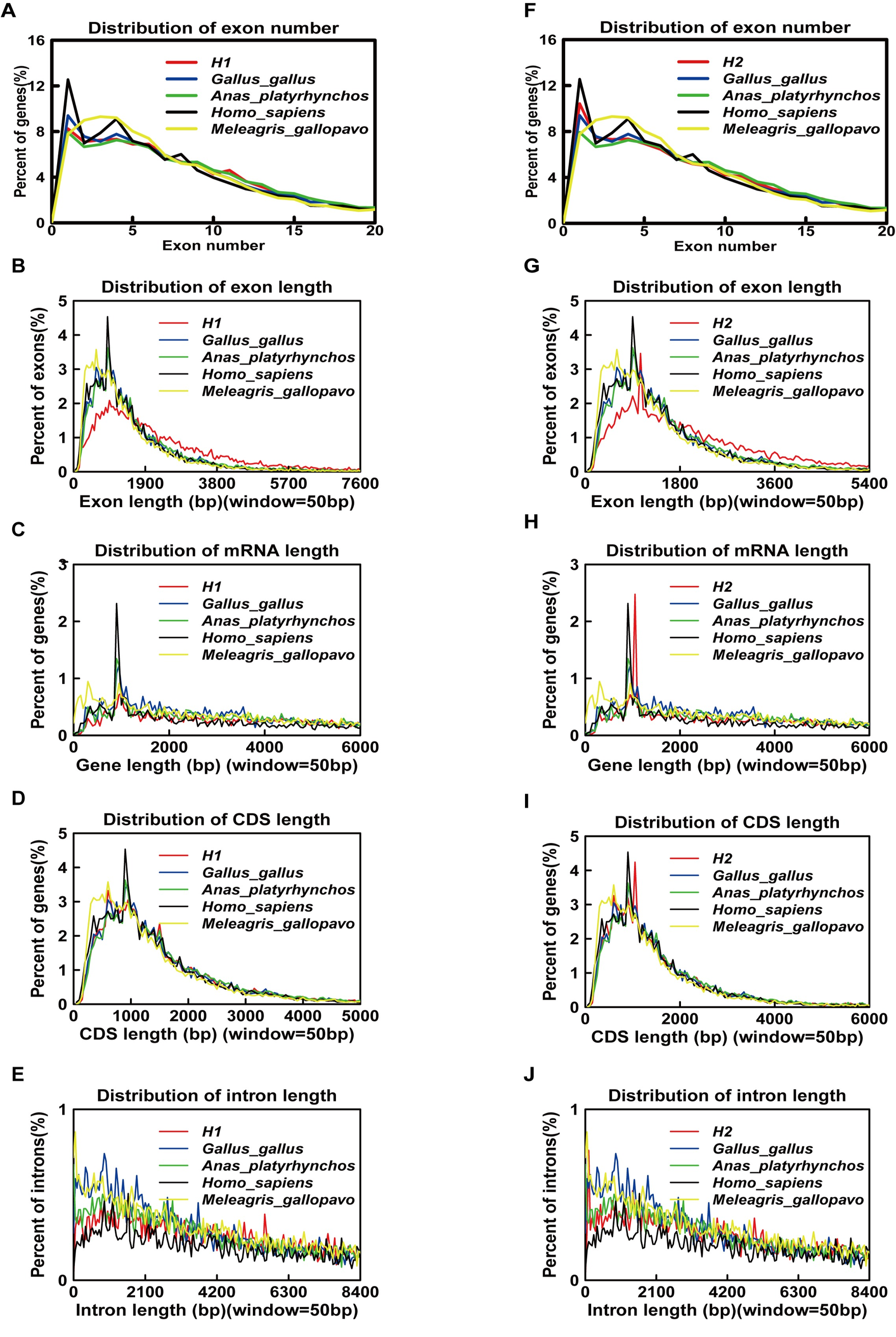

Supplement: S1 File — (ZIP) [file pone.0305914.s001.zip › Supporting_Information/S2_Fig.tif]

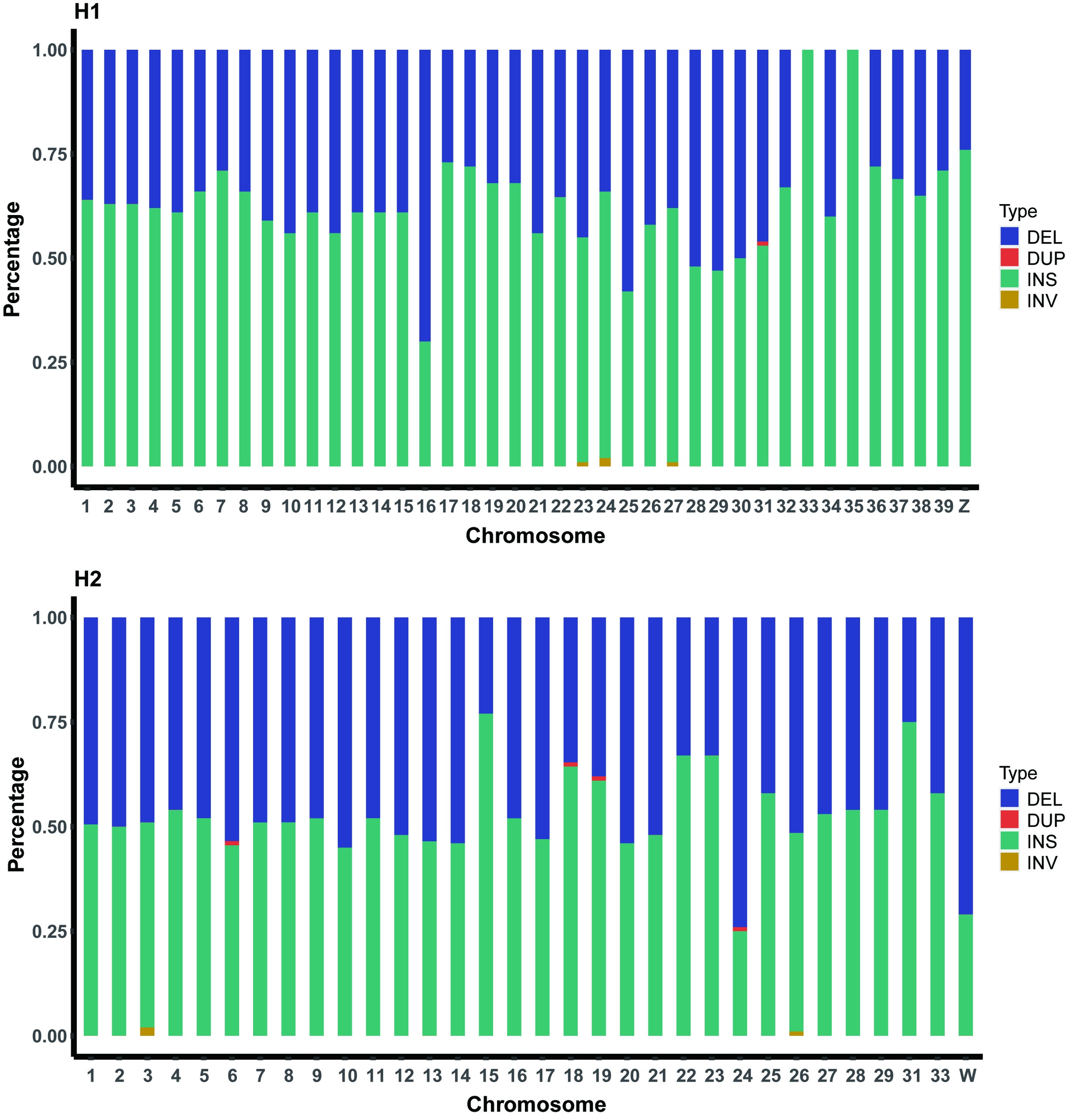

Supplement: S1 File — (ZIP) [file pone.0305914.s001.zip › Supporting_Information/S3_Fig.tif]
